# Interleukin-23 Receptor Gene Polymorphism May Enhance Expression of the IL-23 Receptor, IL-17, TNF-α and IL-6 in Behcet’s Disease

**DOI:** 10.1371/journal.pone.0134632

**Published:** 2015-07-29

**Authors:** Zhengxuan Jiang, Lauren Hennein, Yulin Tao, Liming Tao

**Affiliations:** 1 Department of Ophthalmology, Second Affiliated Hospital of Anhui Medical University, Hefei, Anhui, China; 2 Department of Ophthalmology, University of California San Francisco, San Francisco, California, United States of America; 3 University of California San Francisco School of Medicine, San Francisco, California, United States of America; University of San Francisco, UNITED STATES

## Abstract

**Purpose:**

Recent studies identified an association between Behcet’s disease (BD) and the IL-23R gene polymorphism (rs17375018) in different populations. This study examined whether this IL-23R gene polymorphism is associated with enhanced inflammatory responses.

**Methods:**

We recruited 27 BD patients and 32 controls with three genotypes. Peripheral blood mononuclear cells (PBMCs) were seeded with or without anti-CD3 and CD28. Cells were incubated for 24 hours, and then supernatants were collected and stored at −20◦C until analyzed. Levels of interferon (IFN)-γ, tissue necrosis factor (TNF)-α, interleukin (IL)-17 and IL-6 were detected by ELISA. IL-23R expression was assessed by quantitative real-time polymerase chain reaction (RT-PCR).

**Results:**

The expression of IL-23R was significantly higher in both BD patients and healthy controls with the GG genotype compared to the AG and AA genotype with anti-CD3 and CD28 stimulation (all P-value < 0.05). Among the PBMCs cultured with anti-CD3 and CD28 stimulation, there was an elevated secretion of TNF-α, IL-6 and IL-17 in BD patients and healthy controls with the GG genotype. However, there was no significant change in secretion of IFN- γ in BD patients and healthy controls among the genotype of this IL-23R gene polymorphism.

**Conclusions:**

The results suggest that the GG genotype of the rs17375018 variant in the IL-23R gene enhances pro-inflammatory cytokine responses.

## Introduction

Behçet's disease (BD) is a chronic inflammatory disorder characterized as a recurrent vasculitis and a diverse spectrum of clinical manifestations including recurrent oral aphtha, uveitis, multiform skin lesions, and genital ulcerations [1]. Although the etiology of BD is not fully understood, genetic factors and excessive immune and inflammatory responses play important roles in the pathogenesis of this disease [2,3]. Several genes associated with BD have been identified, but little is known about the detailed mechanisms of these polymorphisms [4,5,6]. It is important to understand whether these particular polymorphisms are associated with enhanced immune and inflammatory responses, as this could play a role in the development of BD.

The IL-23R gene is located on chromosome 1p31, and its relative ligand interleukin-23 (IL-23) is a key component of the immune-regulatory pathway [7]. A recent study identified that the rs17375018 polymorphism in the IL-23R was associated with increased susceptibility to BD, which has been confirmed by subsequent studies in different populations [4,5,8]. The latest study showed that the rs17375018 GG genotype was significantly higher in those with neuro-BD and uveitis [9]. Some studies reported that the production of inflammatory cytokines (IFN-γ, TNF-α, IL-6 and IL17) were significantly elevated in BD patients, and the elevated cytokine levels differed among individuals [3,10]. To examine whether this polymorphism is associated with enhanced inflammatory responses, IL-23R expression and inflammatory cytokine production (IFN-γ, TNF-α, IL-6 and IL17) were investigated in healthy individuals and BD patients with three genotypes of rs17375018. These three genotypes of rs17375018 were found to be associated with BD in our previously published study. [4]

## Materials and Methods

We recruited 27 BD patients with three genotypes (AA n = 8, AG n = 9, GG n = 10), and 32 controls with three genotypes (AA n = 10, AG n = 10, GG n = 12). The blood samples were obtained from the Second Affiliated Hospital, Anhui Medical University (Hefei, China). The diagnosis of BD was based on the criteria of the International Study Group [11]. The study was approved by the local institutional ethics committee of the Second Affiliated Hospital at Anhui Medical University. All procedures were conducted in accordance with the Declaration of Helsinki. The participants provided their written consent in order to participate in this study.

Anticoagulated blood samples were obtained using vacuum tubes containing ethylenediaminetetraacetic acid (EDTA). PBMCs were isolated using Ficoll-Hypaque density gradient centrifugation. While 1 × 10^6^ cells were spared for expression analysis, the remaining cells were seeded at a concentration of 1 × 10^6^ cells/ml into flat-bottom 96-well plates in a complete culture medium with anti-CD3 (1 ug/mL; eBioscience) and anti-CD28 (1 ug/mL; eBioscience). Cells were incubated at 37°C in 5% CO2 for 24 h and then supernatants were collected and stored at -20°C until analyzed. The concentration of IFN-γ, TNF-α, IL-6 and IL17 in the cell culture supernatants was measured using the single analyze ELISA kits (DuoSet ELISA Development; R&D Systems) according to the manufacturer's instructions.

The total RNA from cultured PBMCs and non-cultured PBMCs collected from blood was obtained using Trizol and reverse-transcribed into complementary DNA. IL-23R expression was assessed by quantitative RT-PCR using Taqman assays. The following primers were used: IL-23R forward primer 5’-ACAGTTCCCCAGGTCACATC-3’ and IL-23R reverse primer 5’-CCCAGTTCGGAATGATCTGT-3’. For each sample, the mRNA abundance was normalized to the amount of glyceraldehyde-3-phosphatedehydrogenase (GAPDH).

Data analysis was performed using the ^ΔΔ^Ct method and results were expressed in relative mRNA levels. Expression levels were compared among three groups by the non-parametric Kruskal–Wallis test. Data was expressed as a mean ± SD. A P value < 0.05 was considered statistically significant.

## Results

In this study, there were 27 BD patients (female 13, male 14) and 32 healthy controls (female 16, male 16) with three different genotypes of rs17375018 in the IL-23R, all of which are associated with BD. The average age of the BD patients was 35.8±8.1 years (from 26 to 45 years), and the average age of the healthy controls was 36.4±7.8 years (from 28 to 47 years). The age and gender distribution of the BD patients and controls is shown in Table 1. The age and sex were similar among the different genotype groups in both the BD patients and healthy controls.

**Table 1:** Clinical characteristic of study participants.

Clinical features	Healthy control	Behcet’s disease
Age (years)	36.4±7.8	35.8±8.1
Number	32	27
Sex(F/M)	16/16	13/14
AA genotype	10	8
AG genotype	10	9
GG genotype	10	10

**Fig 1:**
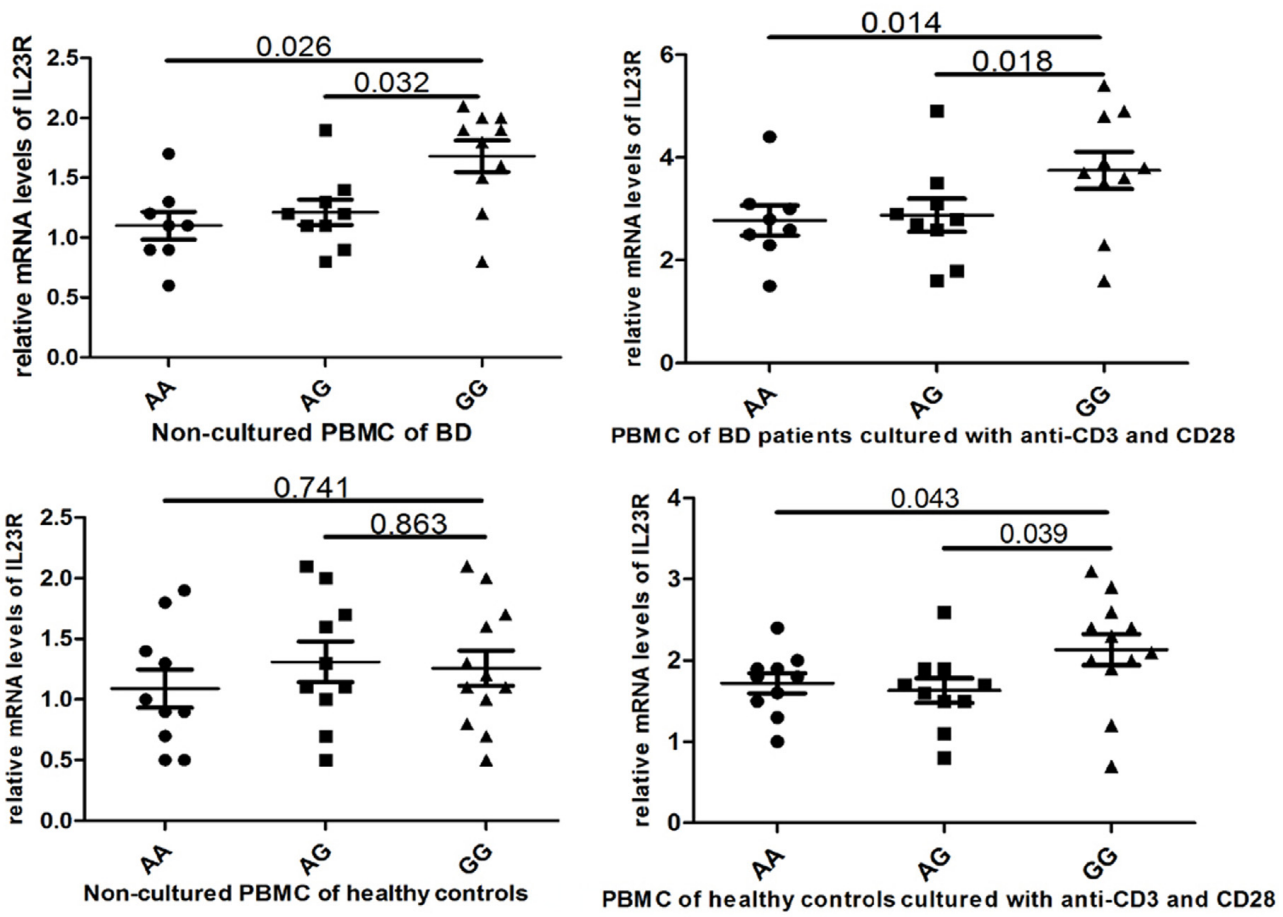
Relative IL-23R subunit expression levels of peripheral blood mononuclear cells (PBMC) obtained from healthy individuals and patients with Behcet’s disease with the rs17375018 AA, AG and GG genotypes.

IL-23R expression levels were comparable among different genotype groups using non-cultured PBMCs, and PBMCs cultured with anti-CD3 and CD28 stimulation. In BD patients, IL-23R expression was significantly elevated in the GG genotype group compared with the AA and AG groups (P = 0.026 and P = 0.032, respectively). After stimulating with anti-CD3 and CD28, the average stimulation index for the GG genotype group was greater than those of AG and AA groups. Furthermore, the GG genotype group showed a significantly higher IL-23R expression level than the other two groups after anti-CD3 and CD28 stimulation (P = 0.014 and P = 0.018, respectively). In healthy controls, the IL-23R expression level was similar amongst the three genotype groups without anti-CD3 and CD28 stimulation (P = 0.741 and P = 0.863, respectively). However, the IL-23R expression in the GG genotype group was significantly higher than the other two groups stimulating with anti-CD3 and CD28 (P = 0.043 and P = 0.039, respectively). The results are shown in Fig 1.

**Fig 2:**
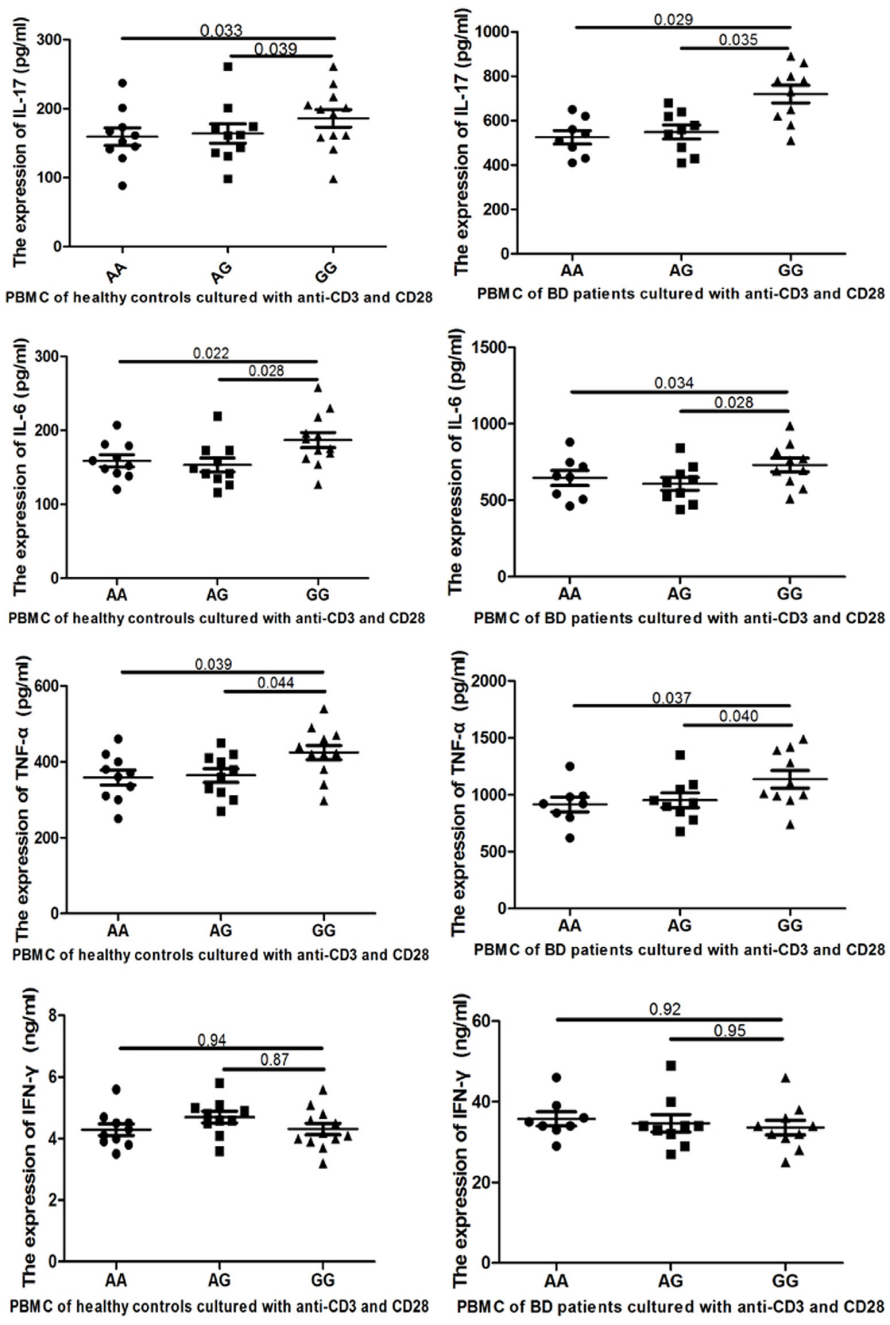
The IL-17, IFN-γ, TNF-α and IL-6 expression levels of peripheral blood mononuclear cells (PBMC) obtained from healthy individuals and patients with Behcet’s disease with the rs17375018 AA, AG and GG genotypes.

IL-17, TNF-α, IL-6 and IFN-γ are important inflammatory cytokines, and are produced by IL-23R signaling [3,8]. In BD patients, the levels of TNF-α, IL-6 and IL-17 were higher in supernatants of anti-CD3 and CD28-stimulated PBMCs from cases with the GG genotype (P = 0.037, P = 0.034 and P = 0.029, respectively), but the level of IFN-γ was similar amongst these three genotype groups (P = 0.920). In healthy controls, the levels of TNF-α, IL-6 and IL-17 were significantly higher in supernatants of the GG genotype individuals with anti-CD3 and CD28-stimulated PBMCs (P = 0.039, P = 0.022 and P = 0.033, respectively). However, although there were elevated trends of IFN-γ in PBMCs stimulated with anti-CD3 and CD-28 in the GG genotype individuals, there was no significant difference among the three genotypes. These results are displayed in Fig 2.

## Discussion

In this study, the rs17375018 risk G allele does not affect the baseline expression level of IL-23R in PBMCs without stimulation, but it enhances IL-23R, TNF-α and IL-17 expression in response to anti-CD3 and CD28 stimulation in healthy controls. In BD patients, the G allele appears to enhance IL-23R production in non-cultured PBMCs and those cultured with anti-CD3 and CD28. In BD patients, the G allele also appears to enhance TNF-α, IL-6 and IL-17 expression capacity in response to anti-CD3 and CD28 stimulation.

Behcet’s disease is a multifactorial autoimmune disease. The prevalence of BD is much higher in the region that extends from China and Japan in the Far East to the Mediterranean Sea, also known as the ancient Silk Route [1,12]. However, BD is rare in Western countries. The etiology of BD is very complex, and it is thought that immunology and genetic factors are both involved in the pathogenesis of BD [3,4,5,8,9,10,13]. Several susceptibility genes, which may regulate the immune reaction, have been found to be associated with BD. However, the precise mechanism of these genes in the development of BD is currently unknown.

This study was designed for the following reasons. First, the IL-23R is essential for the terminal differentiation of IL-17 that produces effecter helper T-cells in vivo [14]. Recent studies have revealed that the rs17375018 G allele was identified to be associated with increased susceptibility to BD in varying populations, but the function of this polymorphism in BD is unknown [4,5,15]. Second, there are many individual differences amongst BD patients due to differences in the severity of disease and the ability of T cells to produce inflammatory cytokines. These results may also be due to single nucleotide polymorphisms (SNPs) in certain genes. Third, the IL-23R generates at least 6 alternatively spliced messenger RNA, leading to diverse isoforms of the receptor proteins. Although the SNPs found to be associated with BD in the IL23-R are synonymous variants or located in noncoding regions, it may influence the function of this gene. These SNPs could affect gene expression through the regulation of transcription factors, changes in mRNA splicing, transport, or stability, and differences in protein folding and stability [16]. In addition, the interaction of IL-23R with its ligand IL-23 stimulates the production of IL-17, TNF-α and IL-6 [17], which are potent pro-inflammatory cytokines that have already been identified to be involved in BD [8,18,19]. Based on these results, the question was therefore raised whether polymorphisms in the IL-23R may influence its function, and thus be associated with pro-inflammatory responses.

Healthy controls were selected to test the differences amongst different genotypes that would not be influenced by disease activity. As the severity of BD may influence the production of inflammatory factors, BD patients were chosen as the study group. On one hand, this study tests whether different genotypes influence the expression of the IL-23R. The results demonstrate that IL-23R expression levels were elevated in the PBMCs of BD patients with the GG genotype, but there was no difference in the PBMCs of healthy controls amongst these three genotype groups. After the PBMCs were cultured with anti-CD3 and CD28, the rs17375018 risk G allele was found to enhance IL-23R expression capacity in response to stimulation in healthy controls and BD patients. These results suggest that the severity of BD may elevate the expression of IL-23R. Similar studies reported that rs924080 in the IL-23R enhances the expression of the IL-23R with lipopolysaccharide stimulation [8]. This study also examined whether different genotypes influence the expression of inflammatory cytokines. Four inflammatory cytokines were selected in this study. IL-17, TNF-α and IL-6 are partially produced by IL-23R signaling, and IFN-γ is produced by IL-12R signaling [10,19,20]. After PBMCs were cultured with anti-CD3 and CD28, the rs17375018 risk G allele was found to enhance TNF-α and IL-17 expression capacity in healthy controls, and TNF-α, IL-6 and IL-17 expression capacity in BD patients. Based on the anti-CD3 and CD28 stimulation results, the rs17375018 G allele may exaggerate inflammatory responses to stimulation, which ultimately leads to BD development. These results are consistent with a previous study which reported that a SNP in the IL-23R elevated the production of TNF-α and IL-6. However, conflicting with our results, the rs924080 risk A allele was also found to enhance the expression of IFN-γ [8].

There were some limitations in our study. The sample size of this study is relatively small, so further studies with a larger sample size are indicated. A previous study performed by Bhakta *et al*. showed that the activity of BD could be considered [13], but our study did not contain this. As the association between disease activity and clinical manifestations are practical and useful, further studies are indicated to identify such trends. The precise mechanism of polymorphisms enhancing the expression of inflammatory cytokines remains unclear, and further studies are indicated. In summary, our results suggest that the GG genotype of the rs17375018 variant in the IL-23R gene seems likely to enhance the expression of the IL-23R and the pro-inflammatory cytokine response.

## Supporting Information

S1 Table The data of the expression about IFN-γ in BD patients and healthy controls. (DOCX)

S2 Table The data of the expression about IL-6 in BD patients and healthy controls. (DOCX)

S3 Table The data of the expression about IL-17 in BD patients and healthy controls. (DOCX)

S4 Table The relative mRNA levels IL23R in BD patients and healthy controls. (DOCX)

S5 Table The data of the expression about TNF-α in BD patients and healthy controls. (DOCX)
