# Supplementary material for: Interleukin-23 Receptor Gene Polymorphism May Enhance Expression of the IL-23 Receptor, IL-17, TNF-α and IL-6 in Behcet’s Disease
Source: PLoS One. 2015 Jul 29;10(7):e0134632. doi: 10.1371/journal.pone.0134632 (PMC4519128; doi:10.1371/journal.pone.0134632)
Supplement: S1 Table — (DOCX) [file pone.0134632.s001.docx]

| healthy controls | | | BD patients | | |
| --- | --- | --- | --- | --- | --- |
| PBMC cultured with anti-CD3 and anti-CD28 | | | PBMC cultured with anti-CD3 and anti-CD28 | | |
| AA | AG | GG | AA | AG | GG |
| 4.12 | 3.58 | 3.29 | 43.20 | 49.30 | 44.10 |
| 3.96 | 3.86 | 3.79 | 38.30 | 40.10 | 38.10 |
| 3.62 | 4.17 | 4.03 | 35.10 | 35.30 | 37.30 |
| 3.85 | 4.21 | 4.01 | 34.10 | 33.70 | 36.70 |
| 4.30 | 4.14 | 4.23 | 33.80 | 34.50 | 35.90 |
| 4.35 | 4.33 | 4.07 | 34.40 | 35.90 | 33.80 |
| 4.43 | 4.56 | 4.34 | 32.70 | 32.40 | 34.50 |
| 4.25 | 4.51 | 4.70 | 28.40 | 27.30 | 34.20 |
| 4.78 | 4.60 | 5.12 |  | 26.10 | 27.40 |
| 5.78 | 5.72 | 5.65 |  |  | 25.70 |
|  |  | 4.24 |  |  |  |
|  |  | 4.19 |  |  |  |

S1 Table. The expression of IFN-γ in BD patients and healthy controls (ng/ml)
