# Supplementary material for: Interleukin-23 Receptor Gene Polymorphism May Enhance Expression of the IL-23 Receptor, IL-17, TNF-α and IL-6 in Behcet’s Disease
Source: PLoS One. 2015 Jul 29;10(7):e0134632. doi: 10.1371/journal.pone.0134632 (PMC4519128; doi:10.1371/journal.pone.0134632)
Supplement: S2 Table — (DOCX) [file pone.0134632.s002.docx]

| healthy controls | | | BD patients | | |
| --- | --- | --- | --- | --- | --- |
| PBMC cultured with anti-CD3 and anti-CD28 | | | PBMC cultured with anti-CD3 and anti-CD28 | | |
| AA | AG | GG | AA | AG | GG |
| 108.00 | 103.00 | 115.00 | 430.00 | 447.00 | 1100.00 |
| 116.00 | 109.00 | 162.00 | 487.00 | 478.00 | 821.00 |
| 124.00 | 114.00 | 139.00 | 519.00 | 521.00 | 793.00 |
| 128.00 | 123.00 | 162.00 | 553.00 | 552.00 | 745.00 |
| 134.00 | 136.00 | 157.00 | 579.00 | 584.00 | 719.00 |
| 125.00 | 132.00 | 168.00 | 617.00 | 603.00 | 692.00 |
| 143.00 | 142.00 | 176.00 | 658.00 | 631.00 | 645.00 |
| 172.00 | 169.00 | 193.00 | 772.00 | 678.00 | 679.00 |
| 175.00 | 175.00 | 197.00 |  | 746.00 | 579.00 |
| 203.00 | 221.00 | 239.00 |  |  | 520.00 |
|  |  | 221.00 |  |  |  |
|  |  | 265.00 |  |  |  |

S2 Table. The expression of IL-6 in BD patients and healthy controls (pg/ml)
