# Supplementary material for: Interleukin-23 Receptor Gene Polymorphism May Enhance Expression of the IL-23 Receptor, IL-17, TNF-α and IL-6 in Behcet’s Disease
Source: PLoS One. 2015 Jul 29;10(7):e0134632. doi: 10.1371/journal.pone.0134632 (PMC4519128; doi:10.1371/journal.pone.0134632)
Supplement: S3 Table — (DOCX) [file pone.0134632.s003.docx]

| healthy controls | | | BD patients | | |
| --- | --- | --- | --- | --- | --- |
| PBMC cultured with anti-CD3 and anti-CD28 | | | PBMC cultured with anti-CD3 and anti-CD28 | | |
| AA | AG | GG | AA | AG | GG |
| 82.00  121.00  112.00  124.00  130.00  148.00  147.00  158.00  190.00  231.00 | 93.00  119.00  110.00  126.00  143.00  137.00  141.00  132.00  188.00  253.00 | 105.00  162.00  143.00  158.00  159.00  179.00  195.00  198.00  213.00  228.00  245.00  273.00 | 687.00  645.00  591.00  578.00  568.00  503.00  472.00  425.00 | 442.00  462.00  494.00  552.00  563.00  598.00  629.00  698.00  667.00 | 820.00  802.00  748.00  723.00  711.00  640.00  609.00  594.00  562.00  501.00 |

S3 Table. The expression of IL-17 in BD patients and healthy controls (pg/ml)
