# Supplementary material for: Interleukin-23 Receptor Gene Polymorphism May Enhance Expression of the IL-23 Receptor, IL-17, TNF-α and IL-6 in Behcet’s Disease
Source: PLoS One. 2015 Jul 29;10(7):e0134632. doi: 10.1371/journal.pone.0134632 (PMC4519128; doi:10.1371/journal.pone.0134632)
Supplement: S4 Table — (DOCX) [file pone.0134632.s004.docx]

| BD patients | | | | | | healthy controls | | | | | |
| --- | --- | --- | --- | --- | --- | --- | --- | --- | --- | --- | --- |
| Non-cultured PBMC | | | PBMC cultured with anti-CD3 and anti-CD28 | | | Non-cultured PBMC | | | PBMC cultured with anti-CD3 and anti-CD28 | | |
| AA | AG | GG | AA | AG | GG | AA | AG | GG | AA | AG | GG |
| 1.75 | 1.85 | 1.98 | 1.65 | 1.81 | 5.42 | 1.88 | 1.95 | 2.09 | 1.83 | 2.64 | 3.02 |
| 1.20 | 1.40 | 1.90 | 2.08 | 1.69 | 5.28 | 1.98 | 2.03 | 2.02 | 1.78 | 1.89 | 2.94 |
| 1.38 | 1.25 | 2.08 | 2.13 | 2.10 | 5.05 | 1.49 | 1.64 | 1.69 | 1.73 | 1.97 | 2.62 |
| 1.23 | 1.30 | 1.79 | 2.21 | 2.15 | 3.95 | 1.41 | 1.52 | 1.60 | 1.62 | 1.74 | 2.46 |
| 1.30 | 1.23 | 1.75 | 2.58 | 2.32 | 3.85 | 1.15 | 1.22 | 1.28 | 2.36 | 1.68 | 2.51 |
| 0.95 | 1.08 | 1.59 | 2.93 | 2.65 | 3.80 | 0.93 | 1.18 | 1.16 | 1.95 | 1.58 | 2.42 |
| 0.90 | 1.14 | 1.48 | 2.64 | 3.01 | 3.72 | 0.90 | 1.12 | 1.18 | 1.45 | 1.47 | 2.31 |
| 0.63 | 0.81 | 1.32 | 4.20 | 2.81 | 3.64 | 0.86 | 1.02 | 1.05 | 1.67 | 1.43 | 2.19 |
|  | 0.92 | 1.26 |  | 4.81 | 2.35 | 0.51 | 0.61 | 0.95 | 1.01 | 1.22 | 2.10 |
|  |  | .81 |  |  | 1.80 | 0.54 | 0.49 | 0.75 | 1.58 | 0.86 | 1.95 |
|  |  |  |  |  |  |  |  | 0.58 |  |  | 1.18 |
|  |  |  |  |  |  |  |  | 0.52 |  |  | 0.74 |

S4 Table. The expression of IL23R in BD patients and healthy controls
