# Supplementary material for: Interleukin-23 Receptor Gene Polymorphism May Enhance Expression of the IL-23 Receptor, IL-17, TNF-α and IL-6 in Behcet’s Disease
Source: PLoS One. 2015 Jul 29;10(7):e0134632. doi: 10.1371/journal.pone.0134632 (PMC4519128; doi:10.1371/journal.pone.0134632)
Supplement: S5 Table — (DOCX) [file pone.0134632.s005.docx]

| healthy controls | | | BD patients | | |
| --- | --- | --- | --- | --- | --- |
| PBMC cultured with anti-CD3 and anti-CD28 | | | PBMC cultured with anti-CD3 and anti-CD28 | | |
| AA | AG | GG | AA | AG | GG |
| 461.00 | 456.00 | 541.00 | 625.00 | 1368.00 | 1490.00 |
| 411.00 | 412.00 | 503.00 | 761.00 | 1123.00 | 1408.00 |
| 387.00 | 402.00 | 483.00 | 798.00 | 1022.00 | 1372.00 |
| 361.00 | 374.00 | 470.00 | 842.00 | 917.00 | 1259.00 |
| 346.00 | 337.00 | 437.00 | 927.00 | 826.00 | 1262.00 |
| 332.00 | 351.00 | 401.00 | 1012.00 | 728.00 | 1081.00 |
| 318.00 | 315.00 | 409.00 | 967.00 | 705.00 | 963.00 |
| 296.00 | 274.00 | 368.00 | 1235.00 | 637.00 | 916.00 |
| 277.00 | 269.00 | 316.00 |  | 602.00 | 892.00 |
| 246.00 | 258.00 | 265.00 |  |  | 751.00 |
|  |  | 420.00 |  |  |  |
|  |  | 418.00 |  |  |  |

S5 Table. The expression of TNF-α in BD patients and healthy controls (pg/ml)
